# Proteomic Analysis of Placental Mitochondria Following Trophoblast Differentiation

**DOI:** 10.3389/fphys.2019.01536

**Published:** 2019-12-20

**Authors:** Joshua J. Fisher, Daniel R. McKeating, James S. Cuffe, Tina Bianco-Miotto, Olivia J. Holland, Anthony V. Perkins

**Affiliations:** ^1^School of Medical Science, Griffith Health, Griffith University, Southport, QLD, Australia; ^2^School of Biomedical Sciences, Faculty of Medicine, The University of Queensland, St Lucia, QLD, Australia; ^3^School of Agriculture, Food and Wine, Robinson Research Institute, University of Adelaide, Adelaide, SA, Australia

**Keywords:** placenta, trophoblast differentiation, mitochondrial isolation, proteomics bioenergetics, mitochondrial metabolism

## Abstract

As gestation proceeds the human placenta is in a constant state of renewal and placental debris is released into the maternal circulation where it can trigger adverse physiological and immunological responses. Trophoblast cells of the placenta differentiate from mononuclear cytotrophoblast cells to fuse and form the syncytiotrophoblast, a multinuclear layer that covers the entire surface of the placenta. As part of this process there are significant changes to cellular cytoskeletal organization and organelle morphology. In this study we have examined the molecular changes that occur in mitochondria from these two cellular compartments and identified differential expression of key proteins that underpin changes in mitochondrial morphology, metabolism and function. Mitochondria were isolated for term placental tissue and separated according to size and density by sequential differential centrifugation. Isolated mitochondrial populations were then subjected to proteomics using HPLC separation of peptides and MS identification. Differential expression of proteins of interest was confirmed by western blots. Using a bioinformatics approach we also examined published protein databases to confirm our observations. In total 651 proteins were differentially regulated in mitochondria from cytotrophoblast versus syncytiotrophoblast. Of these 29 were statistically significant and chosen for subsequent analysis. These included subunits of ATP synthase that would affect ATP production and cristae structure, carbohydrate metabolizing enzymes phospoenolpyruvate carboxykinase-2, pyruvate carboxylase (PC) and pyruvate dehydrogenase (PDH), fatty acid metabolizing enzyme acyl-CoA dehydrogenase, stress responses such a glucose regulated protein-78 and protein disulfide isomerase, and mitochondrial dynamics proteins mitofusin 1 and 2. Placental cell biology and mitochondrial function is central to the pathogenesis of many gestational disorders such as preeclampsia, pre-term birth, fetal growth restriction and gestational diabetes. These studies show important shifts in mitochondrial metabolism and dynamics post trophoblast differentiation and provide key molecular targets for study in pathological pregnancies.

## Introduction

The human placenta is unique in that it is the first organ that is formed and yet it continues to develop and increase in complexity right up until it is discarded 280 days later. Over this period of time, it must continuously adapt to ensure increasing concentrations of energy substrates, micronutrients, and oxygen to the fetus in a timely manner to facilitate appropriate fetal growth and development. It also secretes numerous factors into the maternal circulation to alter systemic hemodynamics, encourage energy substrates to pass to the fetus and prevent immunological rejection of the conceptus. When such processes are perturbed, pregnancy disorders such as preeclampsia, gestational diabetes mellitus and intrauterine growth restriction can result. As such, it is vital to understand appropriate placental development and adaptations to be able to better understand why such pregnancy disorders develop in some women but not in others.

The placenta is derived from the trophectoderm layer of the developing embryo and contains specialized cells called trophoblasts. Proliferation and differentiation of stem cell trophoblasts diverge down two main paths, the extravillous and villous pathway. Extravillous cytotrophoblast play a critical role in placentation, invading and remodeling the maternal spiral arteries to establish a vascular blood supply for the fetus. The villous trophoblast grow to form the placenta and are composed of an underlying villous cytotrophoblast layer which undergoes further differentiation as these mononuclear cells fuse to form a multinucleated syncytium. These two respective cell lineages form the fetomaternal interface which is responsible for the key placental features described above: namely nutrient transport, gas exchange, as well as secretion of immune and endocrine factors into the maternal system. Accompanying the cellular differentiation from cytotrophoblasts to syncytiotrophoblasts are major structural changes to the cytoskeletal network, and in addition their constituent organelles undergo significant structural and functional changes (Jones and Fox, 1991; Martinez et al., 1997).

Placental mitochondria are critical to trophoblast function, undergoing morphological and biochemical transformation post differentiation. Mitochondria from the cytotrophoblast are ovoid or elongated, larger, with clearly defined cristae structure. However, mitochondria in the syncytium appear smaller, spherical and punctate with little to no definition in cristae structure. What drives this process at a molecular level remains unclear although it is known that alterations in morphology are accompanied by changes in mitochondrial bioenergetics, changes in membrane potential and ATP production (Kolahi et al., 2017; Fisher et al., 2019).

The highly dynamic nature of mitochondria as well as the unique morphological and bioenergetic properties of mitochondria from the two major placental cell types has guided this study to better characterize the proteomic differences between cytotrophoblast and syncytiotrophoblast. Furthermore, this study aims to better understand the molecular changes that accompany the transformation of mitochondria following cytotrophoblast differentiation and the role of mitochondria in response to cellular stress in placental pathophysiology. To address this, we have isolated mitochondria from these two cell lineages and used proteomics to explore molecular changes post trophoblast differentiation.

## Materials and Methods

### Mitochondrial Isolation

Ethical approval for all aspects of this research was granted by Queensland Government Human Research Ethics Committee, Australia; HREC/14/QPCH/246 and Griffith University Human Research Ethics Committee; MSC/05/15/HREC.

Isolation of enriched mitochondrial fractions was carried out using sequential differential centrifugation at 1,500 × *g* for 10 min, the supernatant collected and subsequently spun at 4,000 × *g* for 15 min to produce a cytotrophoblast (Cyto-Mito) pellet and 12,000 × *g* to produce syncytiotrophoblast (Syncytio-Mito) pellet as recently described (Fisher et al., 2019).

For this study, all placentae used for mitochondrial isolation and subsequent proteomics (*n* = 3) were from normal healthy pregnancies and were delivered between 39 and 40 weeks gestation, labored and birthed vaginally with no use of drugs to induced labor. For western blotting analysis, the sample set was increased (*n* = 7) with weeks’ gestation expanded to encompass 38–40 weeks to further validate the observations from proteomics with samples.

### Proteomics Following LC-MS Separation

The isolated mitochondrial fractions were prepared for proteomics by liquid chromatography mass spectrometry (LC-MS) as follows. Mitochondrial fractions were lysed in a standardized Urea/Thiourea/Chaps (UTC) buffer, disulfide bonds were reduced with 10 mM dithiothreitol and alkylated with 50 mM iodoacetamide. A further purification step was then performed to enhance protein concentration, separating proteins from detergents, salts, lipids and nucleic acids by precipitation (2-D clean up kit GE Healthcare 80-6484-51). Proteins were then further digested in 6M urea and incubated for 6 h with endoproteinase lysC/trypsin combination digestion mix (1:100, enzyme:substrate) prior to a second digestion (1:50, enzyme:substrate) in 1.5M urea for 18 h. Digested protein samples were run on C18 reverse phase chromatography using a Waters NanoAcquity LC system interfaced to Orbitrap-fusion mass spectrometer (Thermo Fisher Scientific). Peptide separation was achieved over 3 h with 1.5 μg of protein/run. For peptide identification and label-free quantification, the MaxQuant database (Cox and Mann, 2008) was used and cross referenced against the *Homo sapiens* proteome (70,939 canonical sequences, UniProt). This allowed the identification of both mitochondrial and non-mitochondrial proteins. These proteins were then cross referenced against the MitoCarta 2.0 (Broad Institute, United States) database to identify “True” mitochondrial proteins (Pagliarini et al., 2008; Calvo et al., 2016). Cyto-Mito and Syncytio-Mito isolates from three placenta from healthy control pregnancies matched for 39–40 weeks gestation and vaginal delivery were run in triplicate, and proteins with three or more unique peptides sequences were identified. The Bioinformatics platform DAVID^1^ was used to view associations and determine the functional role of the identified proteins. For further validation, a strict criterion was established to determine which proteins would be examined; independent of the fold change, proteins were only examined if they were determined significantly different following a Fisher’s exact test.

### Western Blots

Once proteins of interest for validation had been identified isolates were collected from term placenta (*n* = 7) for examination via western blotting. Protein concentration of mitochondrial isolates were determined using the Pierce BCA Protein Assay Kit (Thermo Scientific, Australia) following the manufacturer’s instructions. 20 μg of protein was loaded onto 12% polyacrylamide gels then separated electrophoretically. Upon completion, proteins were wet-transferred to polyvinylidene fluoride (PVDF) membranes and blocked with Odyssey^®^ blocking buffer. Membranes were incubated with the primary antibody overnight with agitation at 4°C. Primary antibodies utilized in these experiments consisted of β-actin (ab8227) at 1:1000 dilution, ATP5A (ab178421) at 1:1000 dilution, ATPB (ab14730) at 1:1000 dilution, PCK2 (ab187145) at 1:1000 dilution, ACADVL (ab155138) at 1:1000, and GRP78 at 1:1000 (Thermo Fisher PA1-014A). The membranes were washed extensively with Tris Buffered Saline (TBS/Tween) and TBS followed by a subsequent incubation with secondary antibody anti-rabbit (IRDye 680 goat, LI-COR, Lincoln, NE, United States) at 1:1000 dilution, and anti-mouse (IRDye 800CW donkey, LI-COR, Lincoln, NE, United States). The developed blot was then imaged by LI-COR Odyssey and quantified by Image studio v 5.2.

### Pyruvate Dehydrogenase Assay

The level of pyruvate dehydrogenase (PDH) activity was quantified utilizing a PDH enzymatic activity assay (Sigma-Aldrich, Australia) following the manufacturer’s instructions. Samples were run in duplicate and PDH activity is expressed as μmole/min/mg of mitochondrial isolates (*n* = 7).

### Validation of Proteomic Profiles Using Published Single Cell Transcriptome Data

To further validate our findings, an *in silico* approach was used to compare proteomic data from our isolated mitochondria with published data on single-cell RNA sequencing of the human placental trophoblast, which included both cytotrophoblast and syncytiotrophoblast. Pavličev et al. (2017) and Suryawanshi et al. (2018) have recently published RNA transcriptomes from first trimester and term placenta respectively. The data was cross-referenced against MitoCarta2.0 to identify solely mitochondrial changes within these datasets. The independent datasets were submitted to NCBI BioProject under the ID PRJNA492324 and NCBI Gene Expression Omnibus (GEO)^2^ under accession numbers GSE87692 and GSE87726 respectively.

### Statistical Analysis

All values are mean ± SD with Grubbs test for outliers performed. Students *t-*tests were used to determine significance between groups for western blotting (*n* = 7) and enzymatic activity data (*n* = 7). Statistical analysis of the proteomic data was performed using Fisher’s exact test which determines significance based on the average of all unique peptide sequences compared against the background distribution. Significance level was set at 0.05. Analyses were performed using SPSS v22 (IBM SPSS Software, Australia) and GraphPad PRISM 7.02 (GraphPad, United States).

## Results

### Characterizing the Proteomic Profiles of Mitochondrial Subpopulations

As previously published, differential centrifugation resulted in highly enriched populations of cytotrophoblast and syncytiotrophoblast mitochondria (Fisher et al., 2019). Their respective protein profiles were determined via proteomics and results were cross-referenced against the MitoCarta2.0 database to identify solely mitochondrial proteins and enable analysis of varied expression between mitochondrial populations. Of the 649 proteins which matched to MitoCarta2.0, 384 were increased in the Cyto-Mito compared to Syncytio-Mito, 52 remained unchanged, and 213 were increased in the Syncytio-Mito compared to Cyto-Mito. Using significance in Fisher’s exact test, independent to the fold changes as a selection method for further analysis, 29 proteins were identified to have statistically significant different levels of expression between the mitochondrial subpopulations. In total, 24 proteins were expressed at lower levels in Syncytio-Mito compared to Cyto-Mito (Table 1), while 5 proteins were expressed more in Syncytio-Mito compared to Cyto-Mito (Table 2). Following identification of the 29 proteins which met our criteria for further investigation via western blotting and activity assays, the enrichment platform DAVID was used to view associations and determine the functional role of these identified proteins. The proteins can be linked via distinct pathways, with the number of proteins in each pathway as follows; mitochondrial complex subunits (4), pyruvate metabolism (3), fatty acid metabolism (2), amino acid metabolism (9), heat shock proteins (6), antioxidant enzymes (1), mitochondrial aging (1), endoplasmic reticular associated proteins (2), and cristae stabilization (1).

**TABLE 1:** Proteins which were significantly downregulated in syncytiotrophoblast mitochondria compared to cytotrophoblast mitochondria.

**Protein ID**	**Protein name**	**Gene name**	**Average unique peptides Cyto-Mito**	**Average unique peptides Syncytio-Mito**	**Ratio (Cyto/Syncytio)**	**Fold change (Log2ratio)**	**Fishers *p*-value**
**Mitochondrial complex subunits**							
P06576	**ATP synthase subunit beta, mitochondrial**	**ATP5B**	232	136	1.70073	0.766154	7.98*E*−08
P25705	**ATP synthase subunit alpha, mitochondrial**	**ATP5A1**	82	54	1.509091	0.59368	0.009819
P31040	Succinate dehydrogenase [ubiquinone] flavoprotein subunit, mitochondrial	SDHA	41	25	1.615385	0.691878	0.036372
Q9UI09	NADH dehydrogenase [ubiquinone] 1 alpha subcomplex subunit 12	NDUFA12	12	4	2.6	1.378512	0.045514
**Carbohydrate metabolism**							
Q16822	**Phosphoenolpyruvate carboxykinase [GTP], mitochondrial**	**PCK2**	7	0	8	3	0.016184
P11498	Pyruvate carboxylase, mitochondrial	PC	40	17	2.277778	1.187627	0.002017
Q8NCN5	*Pyruvate dehydrogenase phosphatase regulatory subunit, mitochondrial*	*PDPR*	20	9	2.1	1.070389	0.039932
**Fatty acid metabolism**							
P42704	Leucine-rich PPR motif-containing protein, mitochondrial	LRPPRC	71	38	1.846154	0.884523	0.00102
P49748	**Very long-chain specific acyl-CoA dehydrogenase, mitochondrial**	**ACADVL**	101	69	1.457143	0.543142	0.00714
**Amino acid metabolism**							
P12694	2-oxoisovalerate dehydrogenase subunit alpha, mitochondrial	BCKDHA	14	5	2.5	1.321928	0.038312
O15382	Branched-chain-amino-acid aminotransferase, mitochondrial	BCAT2	28	15	1.8125	0.857981	0.046157
P09622	Dihydrolipoyl dehydrogenase, mitochondrial	DLD	55	34	1.6	0.678072	0.019179
P23378	Glycine dehydrogenase (decarboxylating), mitochondrial	GLDC	22	8	2.555556	1.353637	0.009625
P12236	ADP/ATP translocase 3	SLC25A6	38	18	2.052632	1.037475	0.022158
Q02978	Mitochondrial 2-oxoglutarate/malate carrier protein	SLC25A11	38	18	2.052632	1.037475	0.022158
P21397	Amine oxidase [flavin-containing] A	MAOA	263	180	1.458564	0.544548	1.65*E*−05
P30038	Delta-1-pyrroline-5-carboxylate dehydrogenase, mitochondrial	ALDH4A1	47	29	1.6	0.678072	0.028912
**Heat shock proteins**							
P10809	60 kDa heat shock protein, mitochondrial	HSPD1	209	139	1.5	0.584963	4.14*E*−05
P38646	Stress-70 protein, mitochondrial	HSPA9	75	50	1.490196	0.575502	0.015497
Q12931	Heat shock protein 75 kDa, mitochondrial	TRAP1	23	11	2	1	0.038348
**VDAC**							
P21796	Voltage-dependent anion-selective channel protein 1	VDAC1	126	85	1.476744	0.56242	0.002327
P45880	Voltage-dependent anion-selective channel protein 2	VDAC2	78	52	1.490566	0.57586	0.013779
**Antioxidant**							
P04179	Superoxide dismutase [Mn], mitochondrial	SOD2	27	11	2.333333	1.222392	0.022238
**Mitochondrial aging**							
P35232	Prohibitin	PHB	74	52	1.415094	0.500898	0.032229

*Bolded font represents proteins chosen for western blot analysis while italics indicates investigation via activity assay. The ratio derived uses Cyto-Mito as a “normal” mitochondrial variant to assess changes in Syncytio-Mito, so a positive fold change means the protein is greatest in the cytotrophoblast mitochondria, therefore, decreased in the Syncytio-Mito isolates.*

**TABLE 2:** Proteomic analysis of isolated mitochondria from the cytotrophoblast and syncytiotrophoblast cell lineages of the placenta which were expressed significantly greater in syncytiotrophoblast mitochondria.

**Protein ID**	**Protein name**	**Gene name**	**Average unique peptides Cyto-Mito**	**Average unique peptides Syncytio-Mito**	**Ratio (Cyto/Syncytio)**	**Fold change (Log2ratio)**	**Fishers *p*-value**
**Heat shock protein**							
P11021	**78 kDa glucose-regulated protein**	**HSPA5**	93	129	0.723077	−0.46778	0.03683
P14625	Endoplasmin	HSP90B1	94	147	0.641892	−0.6396	0.001886
P07237	Protein disulfide-isomerase	P4HB	47	64	0.738462	−0.43741	0.045503
**Amino acid metabolism**							
P51659	Peroxisomal multifunctional enzyme type 2	HSD17B4	26	40	0.658537	−0.60266	0.04787
**Cristae stabilization**							
Q9NX63	MICOS complex subunit MIC19	CHCHD3	10	22	0.478261	−1.06413	0.020945

*Bolded font represents proteins chosen for western blot analysis.*

### Western Blot and Activity Assay Analysis

**FIGURE 1:**
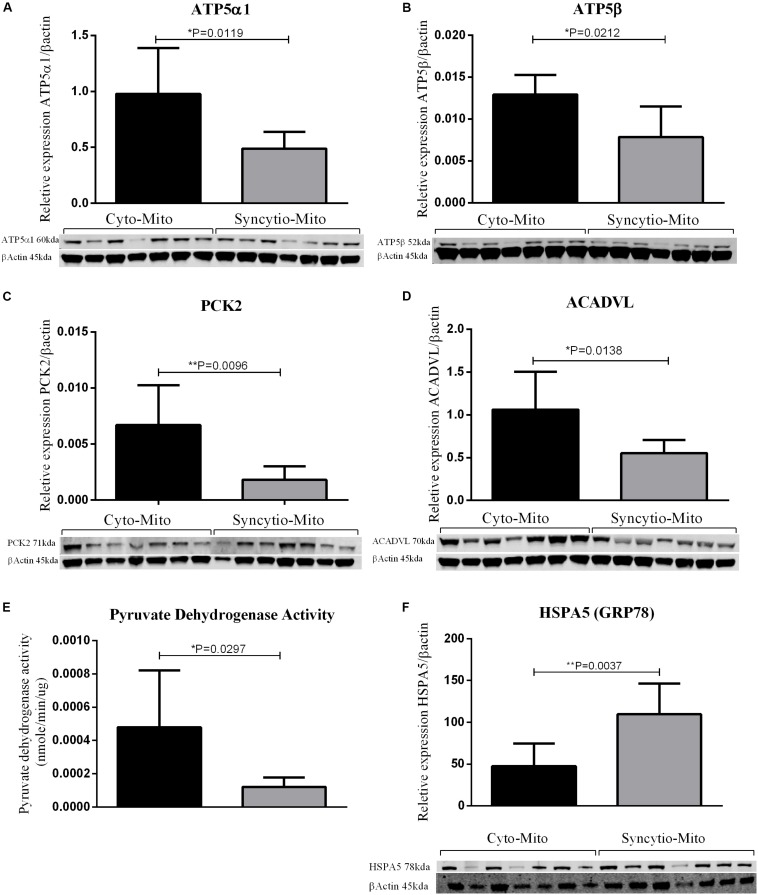
Western blots and densitometry relative to β-actin of Cyto-Mito and Syncytio-Mito for ATP5α1 **(A)**, ATP5β **(B)**, PEPCK2 **(C)**, and ACADVL **(D)**. Pyruvate dehydrogenase activity **(E)** was assessed by activity assay and expressed as nmole/min/μg of protein. Western blots and densitometry relative to β-actin for HSPA5 **(F)** Cyto-Mito (Black), Syncytio-Mito (gray). Mean ± SD, *n* = 7.

To validate findings from the proteomics analysis, western blotting was utilized to examine the expression patterns of 5 proteins and 1 was assessed via an enzymatic activity assay. All findings were consistent with LC-MS proteomic data. Western blot analysis demonstrated that ATP synthase subunit α (ATP5A1-*p* = 0.0119) and ATP synthase subunit β (ATP5B-*p* = 0.0212) expression levels were decreased in Syncytio-Mito compared to Cyto-Mito (Figures 1A,B). PCK2 was selected as a carbohydrate metabolism protein to validate by western blotting analysis. PCK2 expression was lower in the Syncytio-Mito compared to Cyto-Mito (*p* = 0.0096, Figure 1C). PDH protein activity was assessed as a measure of assessing functionality, as PDH is regulated by PDPR which was found to be decreased in the Syncytio-mito when compared to Cyto-mito in the proteomic profile. PDH followed this, with activity found to be greater in the Cyto-Mito compared to Syncytio-Mito (*p* = 0.0297, Figure 1E). Assessment of fatty acid metabolism was also validated by measuring proteins levels of ACADVL which was lower in Syncytio-Mito compared to Cyto-Mito when analyzed by western blot analysis of (*p* = 0.0138, Figure 1D). Of the proteins that increased in the Syncytio-Mito compared to Cyto-Mito, HSPA5 (GRP78) was examined by western blotting which confirmed increased expression (*p* = 0.0037, Figure 1F).

### Validation of Proteomic Profiles Using Published Single Cell Transcriptomic Data

**FIGURE 2:**
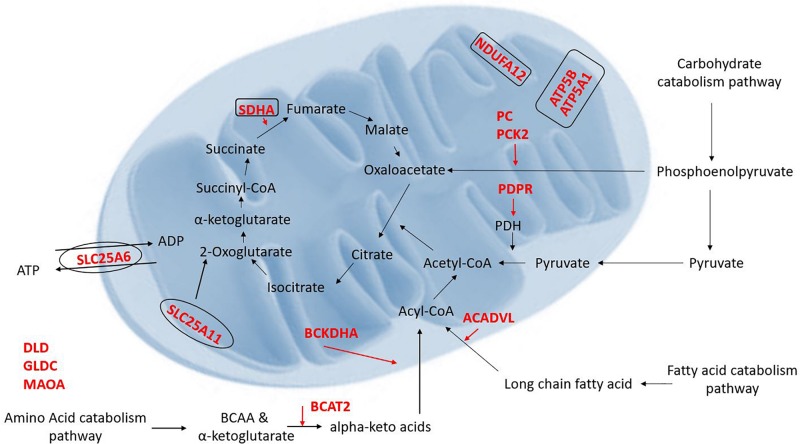
Illustration of the proteins which were found to be decreased (red) in the Syncytio-Mito compared to Cyto-Mito in this study and their implication in mitochondrial function and metabolism (rectangle illustrates mitochondrial complexes, ovals illustrate transporters).

Proteomic results from our Cyto-Mito and Syncytio-Mito samples were compared to single-cell RNA sequencing databases from human placental cell lineages, including syncytiotrophoblast and cytotrophoblasts which were refined to look specifically for MitoCarta 2.0 protein expression within the two cell lineages from the existing datasets (Pavličev et al., 2017; Suryawanshi et al., 2018). The aim of this was to compare our proteomic profiles from isolated mitochondrial populations to that of gene expression of the single cell of origin to determine if the changes we observed at a protein level were conserved in the RNA. Proteomic comparisons of single cell transcriptomics showed a total of 26 genes in total matched our proteomic profiles having altered expression (Table 3). This can be subsequently broken down to investigate the expression matches throughout gestation which showed 18 first and 21 third trimester alterations which matched our proteomic expression changes between our cellular populations. Further comparisons showed 14 genes which matched our proteomic profile were conserved throughout gestation including ATP5A1, SDHA, NDUFA12, BCKDHA, BCAT, and SLC25A6. These proteins have a role across multiple systems and include structural proteins involved in electron transport, proteins associated with carbohydrate, fatty acid and amino acid metabolism, heat shock proteins, mitochondrial ER associated proteins, and antioxidant proteins (see Figure 2). The proteomic analysis presented in Table 1 was performed on term placental mitochondria however first trimester data have been included in Table 3 to show important molecular changes that may relate to stages of gestation. For instance, the down regulation of pyruvate carboxylase (PC) and acyl CoA dehydrogenase (ACADVL), indicating changes in metabolism in the placenta that would influence nutrient supply to the fetus.

**TABLE 3:** Data mining of single cell sequencing analysis of cytotrophoblast and syncytiotrophoblast from first and third trimester placentae which validates the experimental proteomic findings. Expressed as average transcripts per million (TPM).

First trimester					Third trimester				
**Gene**	**CYT**	**SYN**	**Ratio (Cyto/Syncytio)**	**Fold change (Log2ratio)**	**Gene**	**CYT**	**SYN**	**Ratio (Cyto/Syncytio)**	**Fold change (Log2ratio)**
**Mitochondrial complex subunits**									
					ATP5B	389.168	96.36427	4.007301263	2.002631
ATP5A1	10.85863	6.709574	1.618378086	0.69454869	ATP5A1	242.5827	219.799	1.103187599	0.141678
SDHA	1.08374	0.887366	1.22129943	0.288416953	SDHA	25.28696	16.19172	1.529047892	0.612634
NDUFA12	3.312017	1.919608	1.725360662	0.786897968	NDUFA12	130.6539	71.19643	1.823551906	0.866751
**Carbohydrate metabolism**									
PCK2	0.171777	0.036219	4.74273091	2.245718016					
PDPR	0.043427	0.036219	1.199005005	0.261837681					
					PC	3.229057	0	4.229056657	2.080336
**Fatty acid metabolism**									
					ACADVL	232.9049	129.287	1.795304533	0.844229
LRPPRC	1.111726	0.597614	1.860274433	0.895515467					
**Amino acid metabolism**									
BCKDHA	0.36768	0.199205	1.845741039	0.884200154	BCKDHA	19.85554	14.47789	1.347440581	0.430222
BCAT2	0.658157	0.425574	1.54651804	0.629023662	BCAT2	80.94945	0	81.9494509	6.356662
DLD	1.248761	0.74249	1.681856344	0.750054482	DLD	31.23854	8.77372	3.298492153	1.721807
GLDC	0.309778	0.126767	2.443686392	1.28905915	GLDC	103.3204	5.884876	15.15210855	3.921447
					SLC25A6	715.1606	479.5348	1.490340754	0.575642
SLC25A11	1.438874	0.923585	1.557922844	0.639623785	SLC25A11	133.4975	0	134.4975213	7.071436
ALDH4A1	0.673598	0.615723	1.093994109	0.12960497	ALDH4A1	12.14576	1.389207	5.502143902	2.459994
**Heat shock proteins**									
HSPD1	16.14898	14.77736	1.092818614	0.128053963	HSPD1	183.9531	40.5506	4.451273652	2.154218
HSPA9	4.72387	2.60777	1.811459723	0.857152728	HSPA9	85.65878	13.06632	6.1607262	2.6231
TRAP1	1.365531	0.543285	2.513469752	1.329680327	TRAP1	157.0878	4.640674	28.02639676	4.808714
**VDAC**									
VDAC1	5.048123	2.435729	2.07253042	1.051393277	VDAC1	217.4922	69.43148	3.102195366	1.63329
VDAC2	2.148179	1.67513	1.282395263	0.358841002	VDAC2	111.4999	75.27239	1.474975787	0.560691
**Antioxidant mitochondrial aging**									
					SOD2	57.46959	31.50043	1.799040268	0.847227
**Heat shock proteins**									
HSPA5	20.69624	38.23823	0.541244746	–0.88564697					
					HSD17B4	23.51691	51.1044	0.470534	–1.08763
**Cristae stabilization**									
					CHCHD3	38.77691	60.24344	0.649489	–0.62262

## Discussion

Placental mitochondria are critical to organ function and healthy development of the fetus, but little is known about how the unusual Syncytio-Mito are formed from the more stereotypical Cyto-Mito as cellular differentiation occurs, or why the resulting mitochondria are so drastically different in functionality. We found that the levels of 29 proteins were significantly altered between these mitochondrial subpopulations (Tables 1, 2), indicating these proteins may contribute to the unique properties of these mitochondria post cellular differentiation. This included 24 proteins which decreased in expression, and 5 that significantly increased in Syncytio-Mito compared to Cyto-Mito. These proteins are associated with multiple aspects of metabolism, including carbohydrate metabolism, fatty acid metabolism, amino acid metabolism as well as mitochondrial bioenergetics and dynamics. Additionally, there were significant changes in structural proteins involved in mitochondrial electron transport chain (ETC) complex assembly, antioxidant responses, mitochondrial ER tethering, and mitochondrial aging (see Figure 2).

Proteins which are components of ETC complexes ATP5A1 and ATP5B (complex V), SDHA (complex II), and NDUFA12 (complex I) were expressed at significantly lower levels in Syncytio-Mito compared to Cyto-Mito. ATP5A1 and ATP5B make up the α and β subunits of the ATP synthase complex (Jonckheere et al., 2012). Significant decreases in ATPA1 (subunit α, 0.59 fold *p* = 0.0119) and ATPB (subunit β, 0.76 fold *p* = 0.0212) in the Syncytio-Mito were observed by LC-MS proteomics and confirmed by western blotting. The ETC generates ATP, and lower levels of ETC proteins in the Syncytio-Mito may have implications for bioenergetic capacity and ability to supply cellular energy. Indeed, our recently published data on mitochondrial respirometry demonstrated decreased bioenergetic parameters in Syncytio-Mito including; maximum respiratory capacity and ATP production (Fisher et al., 2019). This supports existing literature, which showed altered dimerization of ATP synthase and *F*_1_ to *F*_0_ ratios associated with decreased ATPase activity in mitochondria from Syncytio-Mito and Cyto-Mito (De los Rios Castillo et al., 2011).

Syncytiotrophoblast mitochondria may have reduced respiratory capacity due to additional structural changes in the ETC complexes between cytotrophoblast and syncytiotrophoblast mitochondria. The role of complex I is to transfer electrons from reduced NADH to ubiquinone, leading to translocation of H^+^ from the mitochondrial matrix to the inner membrane space and creating the proton-motive force used by ATP synthesis (Brandt, 2006; Hirst, 2013). NDUFA12 is required for the complete assembly of the matrix arm of complex I and plays a critical role in the NADH dehydrogenase activity of this complex (Rak and Rustin, 2014). Proteomic analysis reported here, found NDUFA12 to be significantly decreased in Syncytio-Mito post differentiation (*p* = 0.0455 and 1.3 fold). This may suggest that Syncytio-Mito lack the correct assembly machinery for a stable and functioning complex I and lack the ability to generate the proton-motive force required to efficiently drive ATP synthase. This structural change could also enhance electron leakage from Complex 1 leading to the excessive generation of reactive oxygen species, a feature of pathological placenta. Additionally, relative lack of this subunit may affect respirasome formation. Respirasomes are super complexes made up of Complex I, III, and IV, and their formation improves the efficiency of the ETC (Rak and Rustin, 2014; Sánchez-Caballero et al., 2016). Therefore, disrupted respirasome formation could lead to reduced bioenergetic efficacy in Syncytio-Mito.

PHB is located in the inner mitochondrial membrane and has been shown to interact with complex I (Bourges et al., 2004). Moreover, PHB deficiency has been shown to decrease respirasome complexes (complex I, III, and IV), which are known to stabilize cristae and effect bioenergetic capacity (Jian et al., 2017). Moreover, studies in neural cells showed overexpression of PHB preserved mitochondrial respiration and elongated mitochondrial morphology (Anderson et al., 2018), similar to the morphology observed in Cyto-Mito and in stark contrast to Syncytio-Mito. In combination, the above significant decreases in ATP5A1, ATP5B, NDUFA12 and associated proteins in syncytiotrophoblast mitochondria strongly suggest a multifaceted and complex mechanistic cause behind the morphological and bioenergetic changes observed in these mitochondria post trophoblast differentiation.

Significant changes in the level of enzymes and proteins involved in the metabolism of carbohydrates, fatty acids and amino acids were also observed in this study. Critical proteins involved in carbohydrate catabolism converging on production of acetyl-CoA were found at different levels in the two mitochondrial populations, including proteins crucial to the conversion of pyruvate to acetyl-CoA such as PDH and the formation of phosphoenolpyruvate a precursor to oxaloacetate. Our research has shown a 2.1 fold decrease in the level of PDPR (PDH phosphatase regulatory subunit) in Syncytio-Mito compared to Cyto-Mito. Mechanistically, PDPR is involved in the phosphorylation and activation of PDH, so is critical to PDH regulation. The functional importance of this finding was confirmed by assessing PDH activity, which was significantly decreased in Syncytio-Mito (*p* = 0.0297). Furthermore, both PC and phophoenolpyruvate carboxykinase-2 (PCK-2) were found to be significantly decreased in Syncytio-Mito (*p* = 0.002, fold change = 1.8 and *p* = 0.016, fold change = 3, respectively). Phosphoenolpyruvate is an important metabolic intermediate in the conversion of pyruvate to oxaloacetate and alterations in the activity of PC and PCK2 would alter the supply of substrates from glucose metabolism to the citric acid cycle (TCA), potentially impairing Syncytio-Mito metabolism (Figure 2). This is supported by a recent publication assessing cytotrophoblasts and the syncytiotrophoblast, which found higher levels of ATP, and greater rates of respiration and glycolysis, in cytotrophoblasts when compared to syncytiotrophoblasts (Kolahi et al., 2017). As gestation proceeds, the underlying cytotrophoblast layer diminishes as it continually fuses to form the syncytium; therefore these metabolic changes may be important to fetal development as they imply that carbohydrate reserves would be utilized less by the placenta as gestation proceeds and be more readily available to the rapidly developing fetus.

Changes in fatty acid metabolism were also observed in this study, converging on the β-oxidation of fatty acids to acetyl-CoA. Very long chain specific acyl-CoA dehydrogenase (ACADVL) was expressed 1.4 fold less in Syncytio-Mito, potentially limiting the first stage of β-oxidation in fatty acids that exceed 16 carbons. Another protein BCKDHA, which impedes acyl-CoA availability, was found to be decreased 1.3 fold in the Syncytio-Mito (*p* = 0.038). BCKDHA is required for the conversion of α-keto acids to acyl-CoA, this process would be further exaggerated through a 0.8 fold decrease in BCAT2 (*p* = 0.046) which converts branched chain amino acids to α-keto acids. Additionally, reduced levels of SDHA (complex II) subunit in the Syncytio-Mito may also be implicated in a decrease oxidative capacity, as the action of this subunit is dependent on the utilization of FAD, a product of β-oxidation which appears to be limited by reductions in multiple key proteins.

Other proteins identified by this study to be decreased in the Syncytio-Mito are involved in amino acid metabolism acting through multiple pathways, including necessary linking enzymes between the urea cycle and the TCA such as ALDH4A1 (1.6 fold *p* = 0.028) which converts proteins to glutamate. Others play more direct roles, such as SLC25A11 (decreased 2 fold, *p* = 0.022), which is integral to the malate-aspartate shuttle and oxoglutarate/isocitrate shuttle by catalyzing the transport of 2-oxoglutarate across the inner mitochondrial membrane. This in conjunction with SLC25A6, transporting free ADP from the cytosol to the inner mitochondrial membrane for exchange of ATP, would all have profound effects on the respiratory capacity and ability of Syncytio-Mito to produce ATP (*p* = 0.022), further supporting existing literature regarding the decrease in ATP producing potential of mitochondria from syncytiotrophoblasts.

This study also found that a small number of proteins were significantly increased in isolated mitochondria from syncytiotrophoblast including HSPA5. HSPA5 is also known as Endoplasmic reticulum chaperone BiP, glucose regulating protein 78 (GRP78) (UniProtKB-P11021) or Heat shock protein 70, and these names refer to the roles of this protein in ER chaperoning. The syncytiotrophoblast is often thought to be a placental region most affected by adverse cellular stresses and chaperones, which assist in stabilizing unfolded proteins, may play and important role in in stabilizing the integrity of the syncytium.

To further validate the findings from these proteomics and western blotting experiments, a meta-analysis was undertaken utilizing multiple single cell RNA sequencing databases, which examined cell lineages from the placenta across gestation (Pavličev et al., 2017; Suryawanshi et al., 2018). This confirmed that many of the proteins identified in the isolated mitochondria from term placenta expressed fold changes in a similar direction. We identified 18 proteins that were different between the isolated mitochondrial populations and confirmed the same RNA expression pattern in the cell lineages from first trimester, increasing to 21 proteins in third trimester including; ATP5A1, ATP5B, SDHA, NDUFA12, PC, and ACADVL. This indicates a mitochondrial adaptation throughout gestation and strongly supports our proteomic findings with 87.5% of proteins identified matching expression patterns from single cell sequencing. We believe this to reflect the mitochondrial subpopulations present in the original datasets as we refined our search using the MitoCarta2.0 database. Further this comparative analysis identified that 14 proteins were higher in the cytotrophoblast than syncytiotrophoblast throughout gestation and again included three structural components of the ETC ATPA1, SDHA, NDUFA12 which may have direct implications on the morphology of the mitochondria as discussed previously. What was evident upon examination of both databases and our proteomic experiments was that syncytiotrophoblast cells and in particular, Syncytio-Mito, have decreased expression of proteins involved in amino acid metabolism with 6 of the 14 proteins conserved throughout gestation involved in these pathways including BCKDHA, BCAT2, DLD, GLDC, SLC25A11, and ALDH4A1. Similarly heat shock proteins appeared to be decreased in the meta-analysis and proteomics across gestation with a further 3 (HSPD1, HSPA9, TRAP1) out of 14 decreased in Syncytio-Mito. This analysis indicates an adaptive ability of mitochondria from the syncytiotrophoblast as gestation progresses, while also clearly highlighting specific structural and functional differences associated which appear to be always present between the two mitochondrial populations.

This study acknowledges the limitations of a small sample size utilized for proteomic analysis, however, this consequently led to the implementation of strict exclusion criteria of three or more unique peptide sequences and the samples being run in triplicate to address this limitation. Further, the unavailability of first trimester placental samples limited the subsequent western blot analysis of proteins identified and suggested to be altered between the mitochondrial populations in early gestation. As evidenced by the *ex vivo* single cell sequencing this is however an important area of study which was confounded by the legality of obtaining tissues from first trimester placenta at the time this study was conducted. Thus, future studies will investigate these changes throughout gestation and the apparent constant alterations between the mitochondria populations from both cell lineages.

Understanding trophoblast biology is key to understanding major gestational complications of pregnancy, and central to this is trophoblast mitochondrial function. Previous studies have indicated that the syncytiotrophoblast is less metabolically active than cytotrophoblasts, and that this might be critical for directing nutrients to the developing fetus whilst maintaining placental homeostasis (Kolahi et al., 2017). This study would support such a hypothesis, as many proteins involved in key stages of carbohydrate, fatty acid and amino acid metabolism are decreased in syncytiotrophoblast mitochondria. Further, many structural proteins involved with complexes of the ETC are decreased in the syncytiotrophoblast, which may have a direct effect on mitochondrial morphology as well as explain the bioenergetic differences reported in the literature and discussed above. The results from this study using isolated mitochondria were confirmed against previous studies that used single cell sequencing analysis of cytotrophoblast and syncytiotrophoblast cell lineages across gestation. This study shows a strong relationship between mitochondrial metabolism and bioenergetics with many of the proteins identified, associated either directly or indirectly with these changes and related to mitochondrial morphology observed in the differentiation of the cell lineages. Many of the changes we have observed in this study suggest that the mitochondrial adaptations in healthy pregnancies occur as a necessary modification to decrease the dependence of syncytiotrophoblasts on carbohydrate, fatty acid and amino acid metabolism. We propose that this is an important adaption to allow adequate supply of nutrients to the fetus during development, as the syncytiotrophoblasts and the organelles within the syncytiotrophoblasts are at the junction between fetal and maternal circulation. The importance of only examining healthy isolated mitochondrial populations in this study will enable a crucial point of reference when addressing how abnormalities in the mitochondria and the resulting dysfunction may underpin pregnancy pathologies.

## Data Availability Statement

The raw data supporting the conclusions of this article will be made available by the authors, without undue reservation, to any qualified researcher.

## Ethics Statement

The studies involving human participants were reviewed and approved by Queensland Government Human Research Ethics Committee, Australia; HREC/14/QPCH/246 and Griffith University Human Research Ethics Committee; MSC/05/15/HREC. The patients/participants provided their written informed consent to participate in this study.

## Author Contributions

JF and DM prepared the placental samples and drafted the first edition of the manuscript. TB-M assisted with the *in silico* analysis which was used to focus on key proteomic changes. JC and OH supervised the laboratory work, assisted in the data analysis, and contributed to the preparation of the manuscript. AP is the PI and was involved in all aspects of the project, and finalized the manuscript for submission.

## Conflict of Interest

The authors declare that the research was conducted in the absence of any commercial or financial relationships that could be construed as a potential conflict of interest.

## Footnotes

1 https://david.ncifcrf.gov/

2 https://www.ncbi.nlm.nih.gov/geo
